# The role of A$\beta$ and Tau proteins in Alzheimer’s disease: a mathematical model on graphs

**DOI:** 10.1007/s00285-023-01985-7

**Published:** 2023-08-30

**Authors:** Michiel Bertsch, Bruno Franchi, Maria Carla Tesi, Veronica Tora

**Affiliations:** 1 Department of Mathematics, University of Roma “Tor Vergata”, Rome, Italy; 2 Istituto per le Applicazioni del Calcolo “M. Picone”, Consiglio Nazionale delle Ricerche, Rome, Italy; 3 Department of Mathematics, Alma Mater Studiorum University of Bologna, Bologna, Italy

**Keywords:** Alzheimer’s disease, Models on graphs, A$\beta$ and Tau proteins, Smoluchowski equations, Numerical simulations, 05C90, 35A01, 35B40, 35Q92, 92C50

## Abstract

In this Note we study a mathematical model for the progression of Alzheimer’s Disease in the human brain. The novelty of our approach consists in the representation of the brain as two superposed graphs where toxic proteins diffuse, the connectivity graph which represents the neural network, and the proximity graph which takes into account the extracellular space. Toxic proteins such as $\beta$ amyloid and Tau play in fact a crucial role in the development of Alzheimer’s disease and, separately, have been targets of medical treatments. Recent biomedical literature stresses the potential impact of the synergetic action of these proteins. We numerically test various modelling hypotheses which confirm the relevance of this synergy.

## Introduction

Alzheimer’s disease (AD) is a neurodegenerative disease characterized by a progressive decline in memory and other cognitive functions, leading ultimately to dementia. According to the 2019 World Alzheimer Report, it is estimated that there are presently 50 million people living with AD and related disorders, and this figure is expected to increase to 150 million by 2050 due to an increasingly aged population.

AD was first described in 1907 by Alois Alzheimer who associated AD with histopathological hallmarks in the brain: senile plaques and neurofibrillary tangles (NFTs). Only after 1980 it was discovered that plaques consist primarily of aggregates of amyloid beta peptides (A$\beta$) (Glenner and Wong 1984), whereas the main constituent of neurofibrillary tangles is misfolded tau protein (Tau) (Grundke-Iqbal et al. 1986). In 1992, Hardy and Higgins (Hardy and Higgins 1992) formulated the so-called amyloid cascade hypothesis for the progression of AD: “the deposition of A$\beta$, the main component of the plaques, is the causative agent of Alzheimer’s pathology and the neurofibrillary tangles, cell loss, vascular damage, and dementia follow as a direct result of this deposition”. Subsequently, this hypothesis has been revised along the years: although senile plaques are associated with AD, their presence is not strictly related to the severity of the disease. High levels of soluble A$\beta$ correlate better with the presence and degree of cognitive deficits. Indeed, diffuse amyloid plaques are commonly present in the brains of cognitively intact elderly people. Some authors (see for instance Haass and Selkoe 2007) overturn the traditional perspective, and claim that large aggregates of A$\beta$ can actually be inert or even protective to healthy neurons. Analogously, A$\beta$ monomers have been shown to lack neurotoxicity (Shankar et al. 2008) and have in fact been suggested to be neuroprotective (Giuffrida et al. 2009; Zou et al. 2002).

In addition, experimental data have shown that the amyloid cascade hypothesis fails to provide a fully satisfactory description of the evolution of AD, since A$\beta$ and Tau seem to act in a synergistic fashion to cause cell death (see, e.g., Ittner and Götz 2011; Ricciarelli and Fedele 2017). On the basis of these results, it has been postulated that, in the AD progression, “A$\beta$ is the trigger and Tau is the bullet” (Bloom 2014).

Thus, though A$\beta$ and Tau remain currently the major therapeutic targets for the treatment of AD (but so far effective therapies are lacking), we shall see in Sect. 2 that recent literature suggests that the interplay between the two proteins should be crucial in the development of the disease and must be taken into account for the development of new therapies that should not be targeted to the two proteins separately. We refer for instance to Bertsch et al. (2021b) for a discussion on the current medical literature.

Mathematical models are the basis for computer simulations, the so-called in silico research which effectively supplements in vivo and in vitro research. An exhaustive historical overview of existing mathematical models for AD up to 2018 can be found in Carbonell et al. (2018). Among more recent contributions to *macroscopic* modeling, we mention (Bertsch et al. 2020, 2021a; Fornari et al. 2019, 2020; Franchi et al. 2020; Goriely et al. 2020; Kevrekidis et al. 2020; Raj et al. 2021; Thompson et al. 2020, 2021; Weickenmeier et al. 2019) and references therein. Several mathematical models, their difficulties, pros and cons are discussed in Bertsch et al. (2021b), where the authors propose a highly flexible mathematical model aimed to take into account as many features of the current research as possible. For a detailed presentation of the connections between this model and various biomedical interpretations, we refer to Bertsch et al. (2021b).

In this Note we present a reduced form of the model in Bertsch et al. (2021b), focused on the study of the interplay of A$\beta$ and Tau and their toxic effect on neurons, though with progressive completeness and complexity. In particular, we face the mathematical difficulty that A$\beta$ is mainly found in the extracellular space and propagates by proximity, while the greater part of Tau is located within the neurons and propagates by neural connectivity (Ahmed et al. 2014) (see Sect. 3).

The core of this paper is the discussion of several numerical simulations, which, thanks to the flexibility of the model, enable us to compare different hypotheses, such as the amyloid cascade hypothesis and different models for the interaction between A$\beta$ and Tau.

Our mathematical model relies on a conceptual scheme that is currently largely accepted in biomedical literature. Roughly speaking, AD is described as a sequence of the following events.

- The first pathological event in AD onset is the overproduction (or the inefficient clearance) of the A$\beta$ peptide. The polymerization of the extracellular toxic A$\beta$ leads ultimately to plaque deposition.
- Toxic A$\beta$ triggers Tau pathology, inducing misfolding of the physiological Tau within neurons and its agglomeration, yielding the formation of toxic polymers and NFT.
- Tau pathology spreads to connected brain regions, driving the neurodegenerative process (we refer for instance to Gallardo and Holtzman 2019, Fig.16.1, Iturria-Medina and Evans 2015 and the references therein).

## Interplay of A$\beta$ and Tau proteins in AD

Let us start by sketching the most relevant properties of the two proteins. At the microscopic level of the neuronal membrane, monomeric A$\beta$ peptides originate from the proteolytic cleavage of a transmembrane glycoprotein, the amyloid precursor protein (APP). By unknown and partially genetic reasons, some neurons present an imbalance between produced and cleared A$\beta$. On the other hand, *macroscopic phenomena* take place at the level of the cerebral parenchyma. The monomeric A$\beta$ diffuses through the microscopic tortuositiy of the brain and undergoes a process of agglomeration, leading eventually to the formation of long, insoluble amyloid fibrils, which accumulate in spherical deposits known as senile plaques (those observed by Alzheimer in his *post mortem* studies).

The Tau protein is mainly found within axons where it stabilizes microtubules, but is also present in smaller amounts in dendrites and in the extracellular space. In AD and other taupathies the Tau protein undergoes two pathological transformations: hyperphosphorylation and misfolding (see e.g. Goedert and Spillantini 2019). In the disease state, the amount of hyperphosphorylated Tau is at least three times higher than that in the normal brain (Iqbal et al. 2010). Hyperphosphorylation of Tau negatively regulates the binding of Tau to microtubules, compromizing microtubule stabilization and axonal transport. It also increases the capacity of Tau to self-assemble and form aggregates from oligomers to fibrils, eventually leading to its deposition as NFTs (Goedert and Spillantini 2017). Furthermore, it has been observed that excess Tau aggregates can be released into the extracellular medium, to be internalized by surrounding neurons and induce the fibrillization of endogenous Tau; this suggests a role for Tau seeding in neurodegeneration (Guo and Lee 2011). This is the so-called mechanisms of release and uptake of the Tau protein.

The soluble monomers and oligomers of the two proteins move in the brain parenchyma. We distinguish two different mechanisms for their diffusion, one for the intracellular Tau inside the connectome (by connectivity, i.e. along neural connections in the brain), and the other one by proximity in the extracellular space for the extracellular A$\beta$.

In AD both A$\beta$ oligomers and misfolded Tau oligomers are known to have a toxic effect on neurons (synaptic dysfunction, neurofibrillary tangle mediated neuron loss, and behavioral deficits), though it is still not well understood which is the precise role of each protein in the progression of the neurodegeneration. In particular, recent studies stress that it is crucial to understand the interplay between the two proteins (see e.g. Busche and Hyman 2020; Ittner and Götz 2011; Kara et al. 2018; Lewis et al. 2001; Pooler et al. 2015). In Ittner and Götz (2011) possible interactions between A$\beta$ and Tau are extensively discussed, suggesting that A$\beta$ drives Tau pathology by causing hyperphosphorylation of Tau, which in turn mediates toxicity in neurons, whereas Tau mediates A$\beta$ toxicity and A$\beta$ and Tau amplify each others toxic effects.

## Parcellation and connectome

The geometric setting of our mathematical model for the interactions between A$\beta$ and Tau must take into account the respective diffusion modes: by proximity (i.e. with a local character) and by connectivity (possibly with a non-local character). Therefore we identify the cerebral parenchyma with a pair of superposed graphs associated with a parcellation of the brain, i.e. a subdivision of the human cerebral cortex into a patchwork of anatomically and functionally distinct areas (parcels).

Following the approach proposed in Raj et al. (2012), we consider a parcellation $\{\Omega _i, \, i=1,\dots N\}$ of the brain and an associated network of white matter fiber pathways connecting these structures. As in Goriely et al. (2020), Raj et al. (2012) and Raj et al. (2021), we represent this network by means a finite weighted graph $G:=\{V,E\}$ in which the vertices $V=\{x_1,\dots ,x_N\}$, are identified with points $x_i\in \Omega _i$, and represent the *i*-th cortical or subcortical gray matter structure (i.e. the *i*-th parcel), while the edges $e_{ij}\in E$ represent the connections by white matter fiber pathways between the *i*-th structure and the *j*-th structure. Coherently, we introduce a family of coefficients $w_{ij}^E\ge 0$ that measure how much the *i*-th structure and the *j*-th structure are connected and are measured by fiber tractography (Raj et al. 2012, 2015). The coefficients $w_{ij}^E$ are said the *connectivity weights* of the graph *G* and we call *G* the “connectivity graph”.

We also consider a second graph $\Gamma :=\{V,F\}$ with the same vertices as *G*, by taking a new family *F* of edges that keep into account the Riemannian distance of the vertices and the heterogeneity of the cerebral parenchyma. More precisely, we assume that two vertices are adjacent if the corresponding parcels of the brain are nearest neighbors. We call $\Gamma$ the “proximity graph” and we associate with $\Gamma$ a family of weights $w_{ij}^F\ge 0$ that take into account the geodesic distance of the *i*-th structure and the *j*-th structure in the cerebral parenchyma. The use of two superposed graphs makes it possible to consider simultaneously the local diffusion of A$\beta$ and the non-local diffusion of Tau. We refer to Remark 1 for a discussion and a comparison with the model in Bertsch et al. (2021a).

The weights $w_{ij}^E$ and $w_{ij}^F$ make possible to introduce the notion of (weighted) Laplacian on *G* and $\Gamma$. More precisely, If $x_m$ is a vertex of *V*, we set

$$\begin{aligned} \pi _{m}^E:= \sum _{j} w^E_{mj}>0\qquad \text{ and }\qquad \pi _{m}^F:= \sum _{j} w^F_{mj}>0. \end{aligned}$$

Associated with the graph *G*, we define the so-called graph Laplacian operator, $\Delta _G$ as follows. Let $x\mapsto g(x)$ be any function defined over the vertices of the graph. Then, for any $m, j\;\text {with}\;1\le m, j \le N$:

1 $$\begin{aligned} \Delta _G g(x_m)=\dfrac{1}{\pi _{m}^E}\sum _{j}( g(x_m)-g(x_j))w_{mj}^E\, . \end{aligned}$$

The graph Laplacian $\Delta _\Gamma$ is defined analogously.

As in Raj et al. (2012, 2021), the map of connectomes can be extracted from a dataset of the MRI of a cohort of healthy subjects and diffusion-weighted MRI (dMRI) scans acquired previously and processed with a custom pre-processing connectomics pipeline.

## The mathematical model

Arguing as in Bertsch et al. (2021b), the mathematical model for the interaction between A$\beta$ and Tau consists of a set of non-dimensional equations on the graphs *G* and $\Gamma$ for the densities of the two proteins, describing their aggregation, diffusion and interactions. Moreover, to describe the evolution of the disease, we introduce a kinetic-type equation for a function *f* meant to describe the health state of the neurons in a fixed parcel $\Omega _i$. Roughly speaking, $f=f(x_i,a,t)$ is the probability density of the degree of malfunctioning $a\in [0,1]$ of neurons located in the *i*-th parcel at time $t>0$ and is such that $f(x_i,a,t)\,da$ represents the fraction of neurons in the *i*-th parcel which at time *t* have a degree of malfunctioning between *a* and $a+da$. For a precise mathematical formulation in terms of probability measures, see Bertsch et al. (2018). We assume that *a* close to 0 stands for “the neuron is healthy” whereas *a* close to 1 stands for “the neuron is dead”. This parameter, although introduced for the sake of mathematical modeling, can be compared with medical images from Fluorodeoxyglucose PET (FDG-PET Mosconi et al. 2010).

**Fig. 1:**
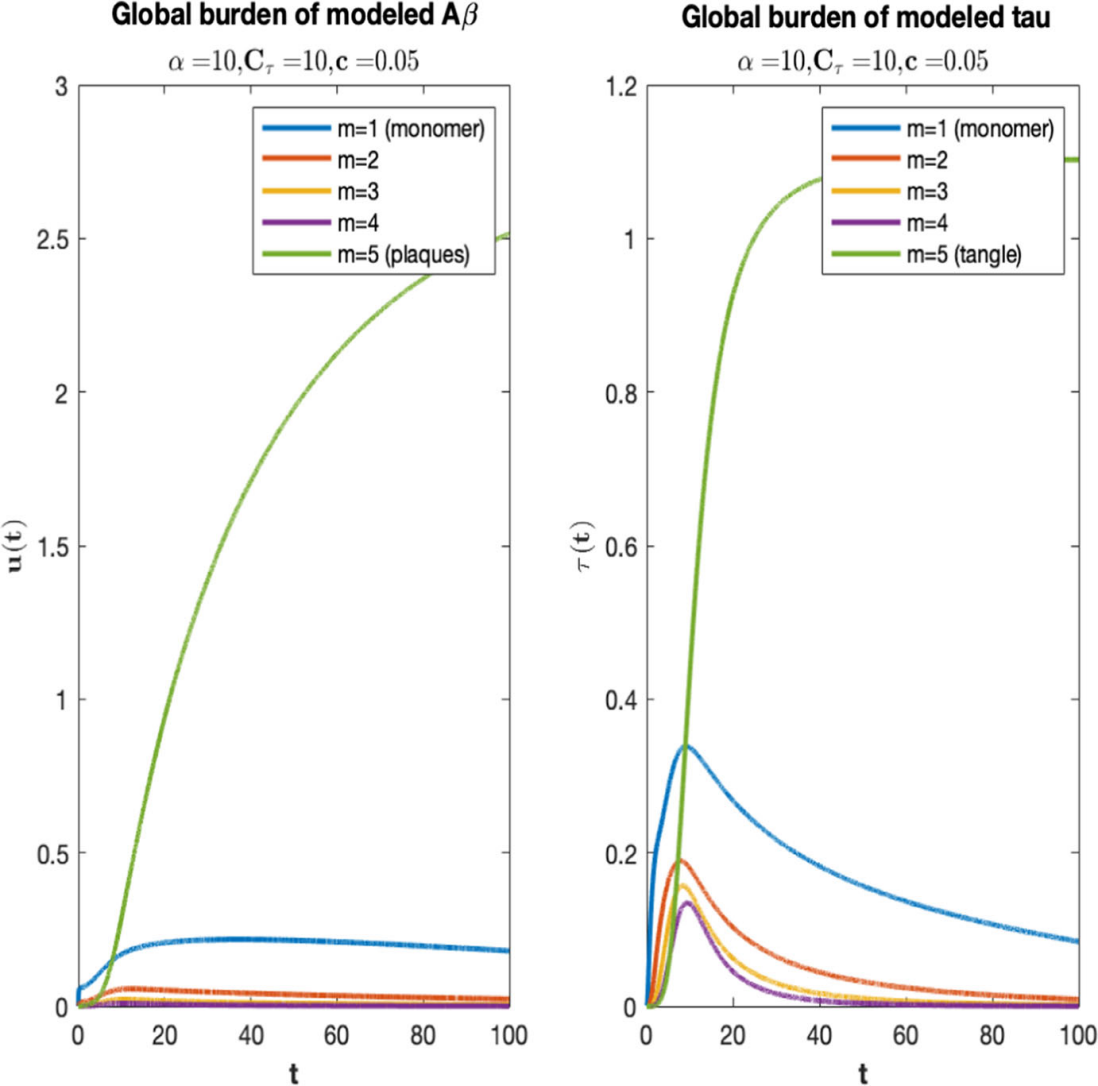
Temporal evolution of the global amount of A$\beta$ polymers (left) and Tau polymers (right) of length $i=1,2,3,4,5$ normalized over all the vertices of the network, where we choose $\alpha =10, C_\tau =10, c=0.05$

In this Note we present a system of reaction diffusion equations to model and test the medical modeling hypothesis described at the very end of the Introduction (see also Fig. 1). The system is a simplification of the diffusion-agglomeration model described in Bertsch et al. (2021b), which is considerably more general but, at the same time, is far too complex to test the medical modeling hypothesis we are interested in. The simplified model enables us to carry on numerical simulations which enlighten the role of mutual relationships of the involved parameters. The general model in Bertsch et al. (2021b) has the same mathematical structure as that presented below, and instead of summarizing it, we list the major simplifications applied in our model. First of all, we completely ignore the presence of Tau in the extracellular space (see, e.g., Guo and Lee 2011) and the strictly related mechanisms of the so-called “release and uptake” of Tau; instead, their contribution to the spreading of the intra-neural Tau is integrated in the diffusion equation within the neuronal network, modeled by the Laplace operator on the connectivity graph. Secondly, instead of considering oligomers of arbitrary length, we divide them in 5 compartments: monomers, dimers, short proto-oligomers, long oligomers and plaques or tangles. All but plaques and tangles may diffuse, whereas plaques and tangles are inert aggregates of molecules of length $\ge 5$.

This is a considerable simplification which strongly reduces the number of parameters in the equations. Finally, we do not take into account fragmentation of polymers and nucleation phenomena (here nucleation means that the concentration of monomers is large enough to trigger the aggregation from monomers into oligomers, the so-called elongation phase).

As for Smoluchowsi’s system, originally, in Smoluchowski (1917) Smoluchowski introduced a system of infinite discrete differential equations (without diffusion and fragmentation) for the study of rapid coagulation of aerosols. Smoluchowski’s theory was successively extended to cover different physical situations. In fact, this type of equations, describing the evolving densities of diffusing particles that are prone to coagulate in pairs, models various physical phenomena, such as, e.g. polymerization, aggregation of colloidal particles, formation of stars and planets as well as biological populations, behavior of fuel mixtures in engines. We refer to Drake (1972) for an historical account.

As far as we know, Smoluchowski’s equations for the description of the agglomeration of A$\beta$ first appears in Murphy and Pallitto (2000) and then in Achdou et al. (2013) and Bertsch et al. (2017a). More recently, different forms of Smoluchowski equations are used in AD models in Bertsch et al. (2020, 2021a), Fornari et al. (2019), Franchi et al. (2020), Franchi and Lorenzani (2016), Franchi et al. (2022), Goriely et al. (2020), Kevrekidis et al. (2020), Raj et al. (2021), Thompson et al. (2021) and Weickenmeier et al. (2019).

Let us sketch qualitatively the arguments leading to these equations: for $k\in {\mathbb {N}}$, let $P_k$ denote a polymer of length *k*, that is a set of *k* identical particles (monomers) that is clustered but free to move collectively in a given medium. Indeed, in biological models, the polymer length *k* is, by its own nature, an integer which does not exceed a certain threshold (this also explains the natural choice of *discrete* Smoluchowski equations). Incidentally, we notice that also continuous Smoluchowski systems have been considered in the literature in different models, for instance when dealing with the so-called polydisperse aerosol: see Drake (1972), Section 2.2.

In the course of time, polymers diffuse and, if they approach each other sufficiently close, with some probability they merge into a single polymer whose size equals the sum of the sizes of the two colliding polymers. For sake of simplicity we admit only binary reactions. This phenomenon is called coalescence and we write formally

$$\begin{aligned} P_k+P_j \longrightarrow P_{k+j} \end{aligned}$$

for the coalescence of a polymer of size *k* with a polymer of size *j*.

Smoluchowski’s equations are presented below. Anyway, throughout this paper, we shall write shortly “Smoluchowski’s equations” for discrete Smoluchowski’s equations with diffusion and without fragmentation. On the other hand, the progression of AD in the *i*-th parcel is determined by the deterioration rate $v=v_i(a,t)$ through a transport equation. More precisely, the equation for *f* has the form (see 10 and 11 below):

2 $$\begin{aligned} \partial _tf+\partial _a(vf)=0 \qquad \text {in } V \times [0,\,1]\times (0,\,T], \end{aligned}$$

where the deterioration rate $v=v_i(a,t)$ depends on the average health state of the neurons in $\Omega _i$ as well on the concentrations of toxic oligomers of A$\beta$ and Tau in $\Omega _i$. A possible simple form of *v*, that nevertheless takes into account the biological phenomena of the disease, is presented in (11).

Thus we are lead to the following system in $V\times (0,\,T]$: if $i=1, \dots , 5$, let us denote by $u_i(x_m,t)$ the molar concentration of A$\beta$ polymers of length *i* at the *m*-th vertex of the graph at time *t*, and by $\tau _i(x_m,t)$ the molar concentration of misfolded Tau polymers of length *i* at the *m*-th vertex of the graph at time *t*. An important role will be played by a small coefficient, $\epsilon >0$, which accounts for two different time scales: a slow one which is typical for the evolution of the disease, and a much faster one which is typical for various physical processes, such as the aggregation and clearance of A$\beta$, and production of A$\beta$ monomers.

If $t>0$ and $x_m\in V$, then the equation for A$\beta$ monomers is

3 $$\begin{aligned} \epsilon \dfrac{\partial u_1(x_m,t)}{\partial t} =\underbrace{- d_1 \Delta _{\Gamma } u_1(x_m,t)}_{\textrm{diffusion}} \underbrace{-\alpha u_1(x_m,t) \sum _{j=1}^{5}u_j (x_m,t)}_\textrm{aggregation} \underbrace{-\sigma _1 u_1(x_m,t)}_\textrm{clearance} \underbrace{ + {\mathcal {F}} (f)}_\textrm{production}\;, \end{aligned}$$

where $\alpha \ge 0$ is the probability of two polymers to coalesce, $\sigma _1\ge 0$ takes into account the clearance of monomers, and ${\mathcal {F}}$ is a source term that will be discussed later.

If $1<i<5$, the equations for oligomers are

4 $$\begin{aligned} \epsilon \dfrac{\partial u_i(x_m, t)}{\partial t}&= \underbrace{- d_i\Delta _{\Gamma } u_i(x_m, t)}_{\textrm{diffusion}} \nonumber \\&\quad \underbrace{+\dfrac{\alpha }{2}\sum _{j=1}^{i-1} u_j(x_m, t) u_{i-j}(x_m,t) -\alpha u_i(x_m, t)\sum _{j=1}^{5} u_j(x_m,t)}_{\textrm{aggregation}}\nonumber \\&\quad \underbrace{-\sigma _i u_i(x_m,t)}_\textrm{clearance}, \end{aligned}$$

where $\sigma _i\ge 0$ takes into account the clearance of oligomers. The diffusion coefficients $d_i$ depend on *i*. For instance, large oligomers have smaller diffusion coefficients.

Finally, the evolution of amyloid plaques is described by the equation

5 $$\begin{aligned} \epsilon \dfrac{\partial u_5 (x_m,t)}{\partial t} =\underbrace{ \frac{\alpha }{2}\sum _{j+k\ge 5; \ k,\,j<5} u_j(x_m,t) u_k(x_m,t)}_{\textrm{aggregation}}. \end{aligned}$$

The lack of diffusion is motivated by the very nature of plaques.

The equation for misfolded Tau monomers at $t>0$ and $x_m\in V$ reads as

6 $$\begin{aligned} \dfrac{\partial \tau _1(x_m,t)}{\partial t}&= - \underbrace{\tilde{d}_1 \Delta _{G}\tau _1 (x_m, t) }_{\textrm{diffusion}} \underbrace{-\gamma \tau _1(x_m,t) \sum _{j=1}^{5} \tau _j(x_m,t)}_{\textrm{aggregation}} \nonumber \\&\quad \underbrace{+c s_{\tau }(x_m,t)}_\mathrm{{production}} + \underbrace{C_\tau \big ( \sum _{i=2}^{4} u_i(x_m,t) -\bar{U}\big )^+}_{\textrm{production}\ \textrm{induced} \ \textrm{by}\ \textrm{A}\beta }, \end{aligned}$$

where $a^+:= \max \{a,0\}$ for all $a\in {\mathbb {R}}$ and, according to Braak staging (Braak and Braak 1991) and denoting by $V_{\textrm{seed}}$ the vertices in the entorhinal cortex, the source term for Tau is $s_{\tau }(x_m, t) = \frac{t}{\lambda }\exp {\left( -\frac{t}{\lambda } \right) }$ for $x_m \in V_{\textrm{seed}}$ and $s_{\tau }(x_m, t)=0$ elsewhere (see also Raj et al. 2021). This Gamma-shaped source term $s_{\tau }$ describes the spontaneous monomer production which first increases in time and then decreases, due to the lack of availability of physiological Tau protein and to the neuronal loss.

In addition, the constant $C_\tau$ modulates the toxic effect of A$\beta$ oligomers on the physiological Tau.

Finally $\gamma$ is the probability of two polymers to coalesce. If $1<i<5$, the equations for oligomers are

7 $$\begin{aligned} \dfrac{\partial \tau _i(x_m,t)}{\partial t}&= \underbrace{- \tilde{d}_i \Delta _{G} \tau _i (x_m,t))}_{\textrm{diffusion}}\nonumber \\&\quad \underbrace{+\dfrac{\gamma }{2}\sum _{j=1}^{i-1} \tau _j(x_m,t) \tau _{i-j}(x_m,t) -\gamma \tau _i(x_m,t)\sum _{j=1}^{5}\tau _j(x_m,t)}_{\textrm{aggregation}} \end{aligned}$$

for $1< i < 5$. Finally, the evolution of tangles is described by the equation

8 $$\begin{aligned} \dfrac{\partial \tau _5 (x_m,t)}{\partial t} =\underbrace{\frac{\gamma }{2}\sum _{j+k\ge 5; \ k,\,j<5} \tau _j(v,t) \tau _k(v,t)}_{\textrm{aggregation}}\;. \end{aligned}$$

As for plaques, the lack of diffusion in (8) is motivated by the very nature of tangles.

We choose the source term ${\mathcal {F}}(f)$ in Eq. (3) as:

9 $$\begin{aligned} {\mathcal {F}}(f)=C_{{\mathcal {F}}}\int _0^1 (\mu _0+a) (1-a)f(x_m,a,t)\;\textrm{d}a \end{aligned}$$

where $f(x_m,a,t)$ is the probability density of the degree of malfunctioning $a \in [0,1]$ of neurons located at the *m*-th cerebral region at time $t>0$. We assume that *a* close to 0 stands for “the neuron is healthy” whereas *a* close to 1 stands for “the neuron is dead”. The aim of the proportionality constant $C_{{\mathcal {F}}}$ is to modulate the amount of A$\beta$ produced by the neuron. When the neuron is healthy ($a=0$) we assume a constant production of A$\beta$ monomers represented by the constant $C_{{\mathcal {F}}}\mu _0$. The term $(1-a)$ is meant to take into account that a dead neuron ($a=1$) does not release A$\beta$. Remember that $\int _0^1 f(x_m,a,t)\;\textrm{d}a=1$, since $f(x_m, \cdot , t)$ is a probability density.

The function $f(x_m,a,t)$ satisfies:

10 $$\begin{aligned}&\partial _t f(x_m,a,t) + \partial _a \big ( v[f(x_m,a,t)] f(x_m,a,t)\big )=0\nonumber \\&f(x_m,1,t)=0 \;\quad \text {for any}\; m =1,\dots , N, t \ge 0 \nonumber \\&f(x_m,a,0)=f_{0}(x_m,a) \;\quad \text {for any}\; m =1,\dots , N, \end{aligned}$$

where

11 $$\begin{aligned} v[f(x_m,a,t)]&= C_{{\mathcal {G}}}\int _0^1 (b-a)^+ f(x_m,b,t)\;\textrm{d}b +C_S (1-a) \big ( \sum _{i=2}^{4}u_i(x_m,t) -\bar{U}_{A\beta } \big )^+\nonumber \\&\quad + C_T (1-a) \big ( \sum _{i=1}^{5}\tau _i(x_m,t) -\bar{U}_{\tau } \big )^+\;. \end{aligned}$$

Here, the first term in the right-hand side describes the possible prion-like propagation of AD through the neural pathway. Malfunctioning neighbors are harmful for a neuron’s health state, while healthy ones are not. In addition, the terms $C_S (1-a) \big ( \sum _{i=2}^{4}u_i(x_m,t)-\bar{U}_{A\beta } \big )^+$ and $C_T (1-a) \big ( \sum _{i=1}^{5}\tau _i(x_m,t)- \bar{U}_{\tau } \big )^+$ represent the toxic effect of $A\beta$ and Tau, respectively. The constants $\bar{U}_{A\beta }$ and $\bar{U}_{\tau }$ are positive threshold values below which the toxic proteins fail to be harmful.

### Remark 1

We stress that the present model differs from the one presented in Bertsch et al. (2021a), since here the diffusion of Tau takes place along the edges of a weighted graph, while in Bertsch et al. (2021a) we have an isotropic diffusion in the cerebral parenchyma.

## Numerical simulations and discussions

First of all, in this Section we present several numerical simulations of the mathematical model introduced in Sect. 4. Subsequently, we shall discuss variants of this model corresponding to different scenarios that have been considered in the biomedical literature.

As in Raj et al. (2012, 2021), the map of connectomes is extracted from a dataset of the MRI of a cohort of healthy subjects and diffusion-weighted MRI (dMRI) scans acquired previously and processed with a custom pre-processing connectomics pipeline. In our simulations, we retrieve the data from Szalkai et al. (2015, 2017), as well as the web site https://braingraph.org.

To perform numerical simulations, the choice of parameters is a crucial issue. Since the data available in the medical literature do not provide uniquely all the values we need, and, in addition, our model is meant to be descriptive and not predictive, we perform arbitrary but realistic choices. By “realistic” we mean that the outputs of our numerical simulations are in some way comparable with clinical data. The parameters that are kept fixed are summarized in Table 1.

**Table 1:** Values of the fixed parameters

*N*	$d_i=\tilde{d}_i$	$\sigma _i$	$\epsilon$	$\gamma$	$\lambda$	$\bar{U}$	$C_{{\mathcal {G}}}$	$C_S$	$C_T$	$\bar{U}_{A\beta }$	$\bar{U}_{\tau }$	$C_{{\mathcal {F}}}$	$\mu _0$
1015	1/i	1/i	0.1	4	10	0.001	0.1	0.01	0.01	0.001	0.001	10	0.01

The remaining parameters will be discussed in Sect. 5.3, testing various hypotheses concerning the progression of neurodegenerative processes associated with different choices of the constants $\alpha , C_{\tau }, c$. These parameters control respectively the A$\beta$ agglomeration, the production of monomeric Tau driven by A$\beta$ oligomers and the Tau seeding at the entorhinal cortex (modeled through the Gamma-shaped functions $s_{\tau }$).

As for the initial data, we choose $$f_0(x_m,a) $$ of the form of a function approximating the Dirac delta centered in a point *a* close to 0. Thus $$f_0$$ represents an (almost) healthy brain. Moreover, again in the spirit of starting from a healthy brain, we choose

12 $$\begin{aligned}&u_1(x_m,0)= u_{0,1}(x_m)<<1 \; \text {for any}\; x_m \in V\nonumber \\&u_{i}(x_m,0)=0\; \text {for any}\; x_m \in V, \;2\le i \le 5\nonumber \\&\tau _i(x_m,0)=0 \; \text {for any} \;x_m \in V, \;1\le i \le 5\;. \end{aligned}$$

In order to provide global pictures of the evolution of the disease in the brain (or in large portions of the brain), we introduce some macroscopic quantities (the total burden of A$\beta$ and Tau and the average degree of malfunctioning): assuming that all parcels have the same volume, the global amounts of A$\beta$ and Tau polymers are given (up to dimensional constants) by

13 $$\begin{aligned}&u_i(t) =\frac{1}{N}\sum _{x_m \in V} u_{i}(x_m,t) \; \text {for}\;1\le i \le 5\nonumber \\&\tau _i(t)=\frac{1}{N}\sum _{x_m \in V}\tau _i(x_m,t) \; \text {for} \;1\le i \le 5\;, \end{aligned}$$

where we recall that *N* is the number of vertices of *G* and $\Gamma$. The evolution of the disease is described in each node of the brain network by the local average of the degree of malfunctioning of the neurons:

14 $$\begin{aligned} A(x_m,t)=\int _0^1 a f(x_m,a,t) \textrm{d}a \;\text {for} \;x_m \in V\;. \end{aligned}$$

Thus, the evolution of the disease in the whole brain in given by:

15 $$\begin{aligned} A(t)=\frac{1}{N}\sum _{x_m \in V} A(x_m,t), \end{aligned}$$

### Total burden of A$\beta$ and Tau in the whole brain

In Fig. 1, we plot the longitudinal graphs of $u_i(t)$ and $\tau _i(t)$ (see 13) with $\alpha =10$, $C_\tau =10$, and $c=0.05$

For both proteins, monomers’ curves are the first to rise followed by those of the oligomers in increasing length. Each monomeric and oligomeric curve peaks and subsequently begins to decrease. This corresponds to the clinical experience of advanced AD (see, e.g. Ballard et al. 2011). Moreover, low concentration of A$\beta$ in CSF (Celebral Spinal Fluid) is listed among diagnostic criteria and differential diagnosis of Alzheimer’s disease from other dementias. This behavior is consistent with several factors. First, as for the A$\beta$, the imbalance between the source ${\mathcal {F}}(f)$ and the clearance leads monomers to increase, while concerning Tau protein, the monomers’ growth is due to the seeding at EC and the effect by A$\beta$ oligomers. The first process drives the production of misfolded tau monomers at earlier times but then declines compatibly with the fact that the available pool of cleavable protein is limited, due to the loss of neurons. On the other hand, the production of misfolded Tau monomers, governed mainly by the effect of by A$\beta$ oligomers is lagged in time with respect to Gamma-shaped seeding since oligomers have to reach a certain amount to damage the neuron. Clinical evidences of the time lag between the presence of A$\beta$ and Tau in the CSF are known in medical literature, see e.g. Jack et al. (2013).

The coagulation process described by means of Smoluchowski equation causes progressively the formation of larger oligomers leading to insoluble aggregates, taking active oligomers out of circulation. Insoluble clusters (plaques and tangles) are the last to develop and their curves show an increase while oligomers decrease.

### Local burden of A$\beta$ and Tau in different regions

The aim of this Section is to simulate the progress of the formation of oligomers, plaques and tangles in different regions of the cerebral parenchyma. To this end, we localize the total burden functions of (13) into different regions as follows. Let $\tilde{R}=\{ R_1, \dots , R_{\ell } \}$ be a finite partition of *V*, where each subset $R_{j}$ can be considered as a region of the brain. Thus, for the *j*-th region, setting $r_j=|R_{j}|$, we define:

16 $$\begin{aligned}&u_{i,R_j}(t) =\frac{1}{r_j}\sum _{x_m \in R_j} u_{i}(x_m,t) \; \text {for}\;1\le i \le 5\nonumber \\&\tau _{i,R_j}(t)=\frac{1}{r_j}\sum _{x_m \in R_j}\tau _i(x_m,t) \; \text {for} \;1\le i \le 5\;. \end{aligned}$$

17 $$\begin{aligned}&A_{R_j}(t)=\frac{1}{r_j}\sum _{x_m \in R_{j}} A(x_m,t). \end{aligned}$$

**Fig. 2:**
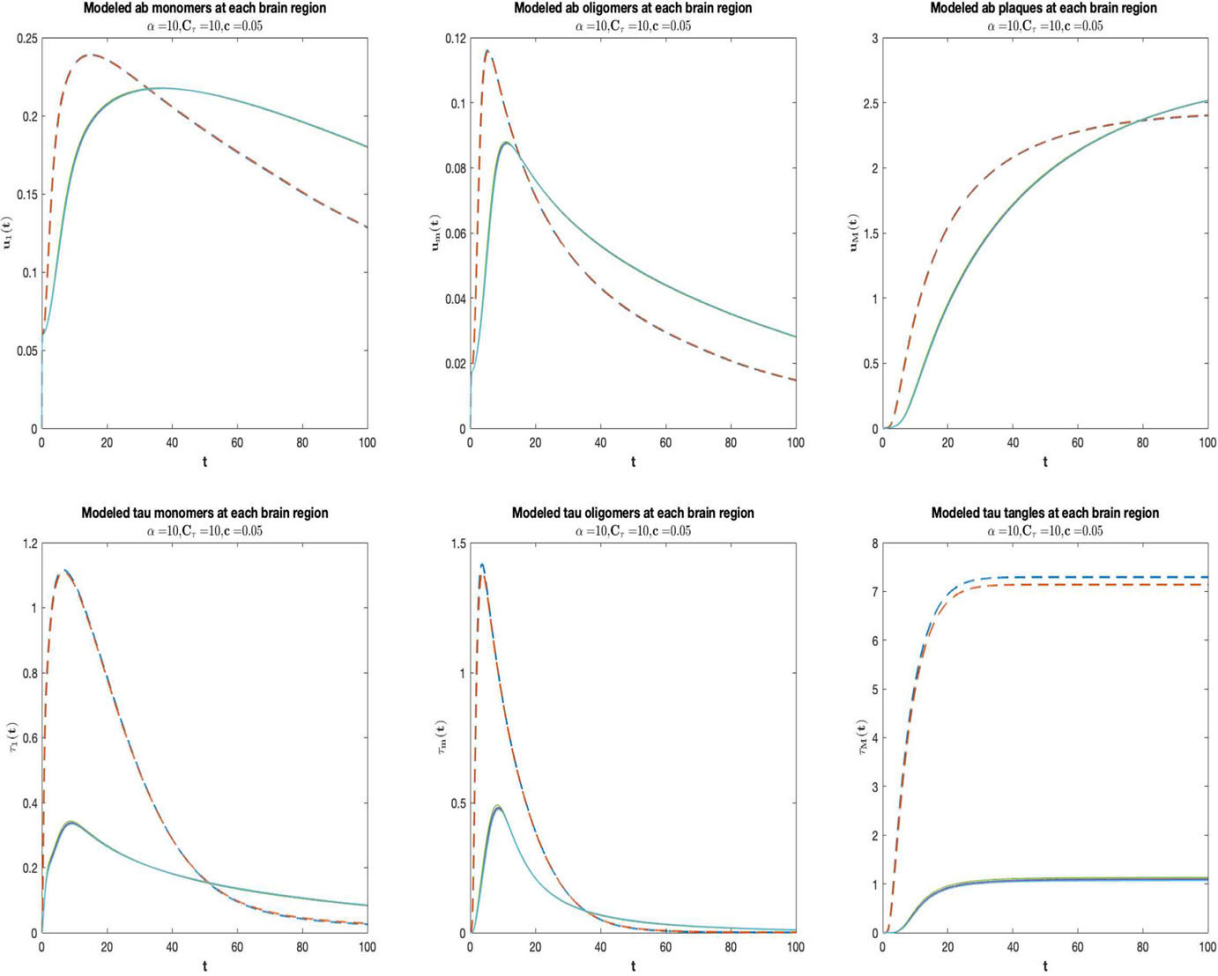
Temporal evolution of A$\beta$ (top) and Tau (bottom) monomers (left), oligomers (middle) and tangles (right) at each brain region for $\alpha =10, C_\tau =10, c=0.05$. Each curve corresponds to a distinct region. The dashed curves represent the right entorhinal cortex (blue) and the left entorhinal cortex (red) (color figure online)

The evolution in time of monomeric, oligomeric and insoluble clusters of A$\beta$ and Tau proteins in each brain region are shown in Fig. 2 for $\alpha =10$, $C_\tau =10$, and $c=0.05$. Each curve corresponds to a distinct region. The dashed lines indicate the regions of the entorhinal cortices (EC), more precisely the right entorhinal cortex and the left entorhinal cortex. Consistently with the plots in Fig. 1, monomers and oligomers’s regional curves rise, peak and subsequently begin to decline while plaques and tangles’ regional curves increase until they reach a plateau. Longitudinal graphs for different brain’s regions of both A$\beta$ and Tau proteins overlap, except for the entorhinal cortex. Here, as expected, the concentrations of both soluble A$\beta$ and soluble Tau are bigger than in other brain areas at earlier times. Indeed, in the EC monomeric Tau and A$\beta$ rise from zero faster and consequently oligomers as well as insoluble clusters form earlier than in other brain areas. While the plaques burden in EC is bigger at least at earlier times, the tangle burden in EC is more sizable than in other areas during all the course of the disease. Although regional differences in the distribution of A$\beta$ and Tau distribution as well as in the disease’s evolution are not very evident except than in the seeding zone, small variations in A$\beta$ and Tau burdens are present. Next, the regions near the entorhinal cortex where the burden of A$\beta$ and Tau clusters is more considerable, are (in order): amygdala, hippocampus, temporal pole, isthmus of cingulate cortex, insula cortex and parahippocampal cortex). Consistently, in these regions the evolution of the disease is more severe.

### Global and regional behavior under different medical hypotheses

We then test various hypotheses concerning the progression of neurodegenerative processes which are associated to different choices of the constants $\alpha , C_{\tau }, c$. We recall that these parameters control respectively the A$\beta$ agglomeration, the production of monomeric Tau driven by A$\beta$ oligomers and the Tau seeding at the entorhinal cortex.

**Table 2:** Simulated cases and respective values of parameters

	$\alpha$	$C_\tau$	*c*
Case A	10	0	0
Case B	10	0	0.05
Case C	10	10	0.05
Case D	10	10	0
Case E	0	10	0.05

The situations considered are listed in Table 2. In case A the sources of Tau monomers are taken out; hence, Tau protein is not produced. This situation might represent a simplified amyloid cascade hypothesis. In cases B and D, we remove, one by one, the sources of Tau monomers, represented respectively by A$\beta$ oligomers and by Tau seeding at EC, while in case C all these processes are included. Notice that case C has already been considered in Sects. 5.1 and 5.2. In case E, we inhibit the agglomeration of A$\beta$ in clusters and consequently, the process of Tau misfolding driven by A$\beta$ oligomers is not triggered independently from the value of the constant $C_\tau$. This might represent a possible effect of a drug.

In Fig. 4, longitudinal graphs of $A_R(t)$ are shown in each of cases summarized in Table 2. The curves increase in time in each brain region, consistently with the fact that the cerebral damage grows as long as the AD pathology progresses. Each curve indicate a distinct region. The dashed lines correspond to the regions of the entorhinal cortices (more precisely the right entorhinal cortex and the left entorhinal cortex) where Tau misfolding is seeded. In case A, the neuronal damage is mild and roughly homogeneous in all regions, thus, the disease progression is slow almost everywhere. In case B, the injury is not critical in most of the cerebral areas, but, compared to case A, it is much more serious at the areas of the entorhinal cortex, where monomeric Tau is seeded. Compared to cases A and B, in cases C and D the evolution of the disease is more severe in all cerebral regions, and, in particular, in case C the EC is the most damaged brain area. Case E resembles case B, since the harm is moderate in all regions, except in the EC. In such regions, the AD pathology starts and its progression in time is more severe than in other areas of the brain.

**Fig. 3:**
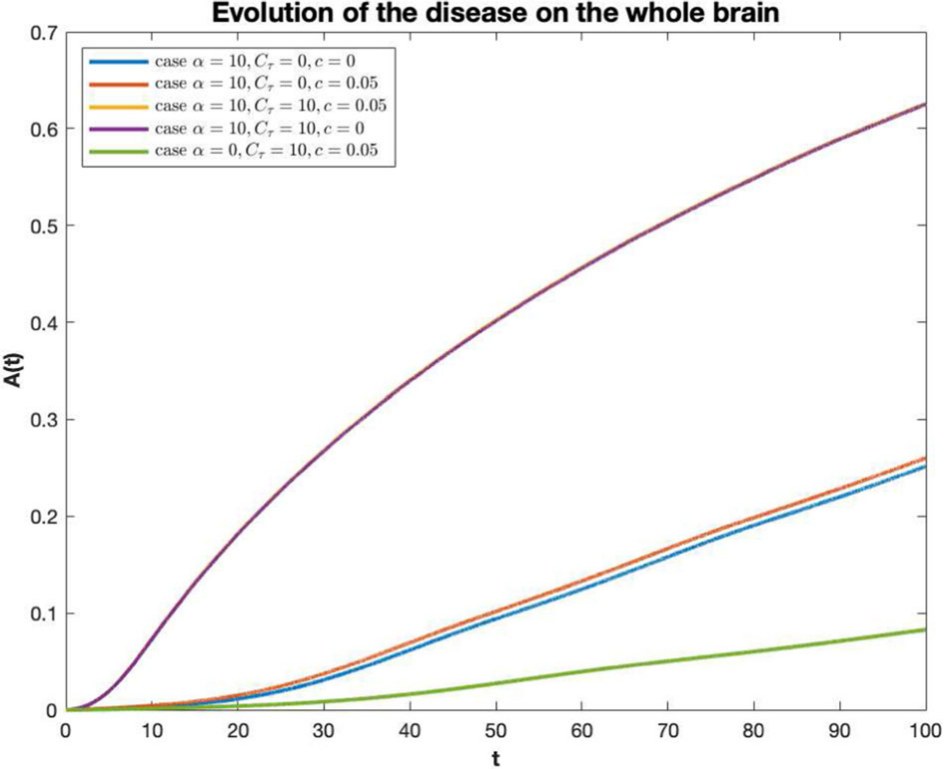
Temporal evolution of the AD in the whole brain for $\alpha =10, C_\tau =0, c=0$, (blue curve), $\alpha =10, C_\tau =0, c=0.05$, (red curve), $\alpha =10, C_\tau =10, c=0.05$, (purple curve), $\alpha =10, C_\tau =10, c=0$, (yellow curve), $\alpha =0, C_\tau =0, c=0.05$, (green curve) (color figure online)

It is interesting to observe that when the production of monomeric Tau driven by oligomeric A$\beta$ is neglected (cases B and E), the neuronal damage is mild and localized to some brain areas. As expected, it is serious in the region of entorhinal cortex. On the other hand, when the oligomeric A$\beta$ directly induces the production of monomeric Tau (case C and D), the evolution of the disease is severe in all the cerebral regions.

**Fig. 4:**
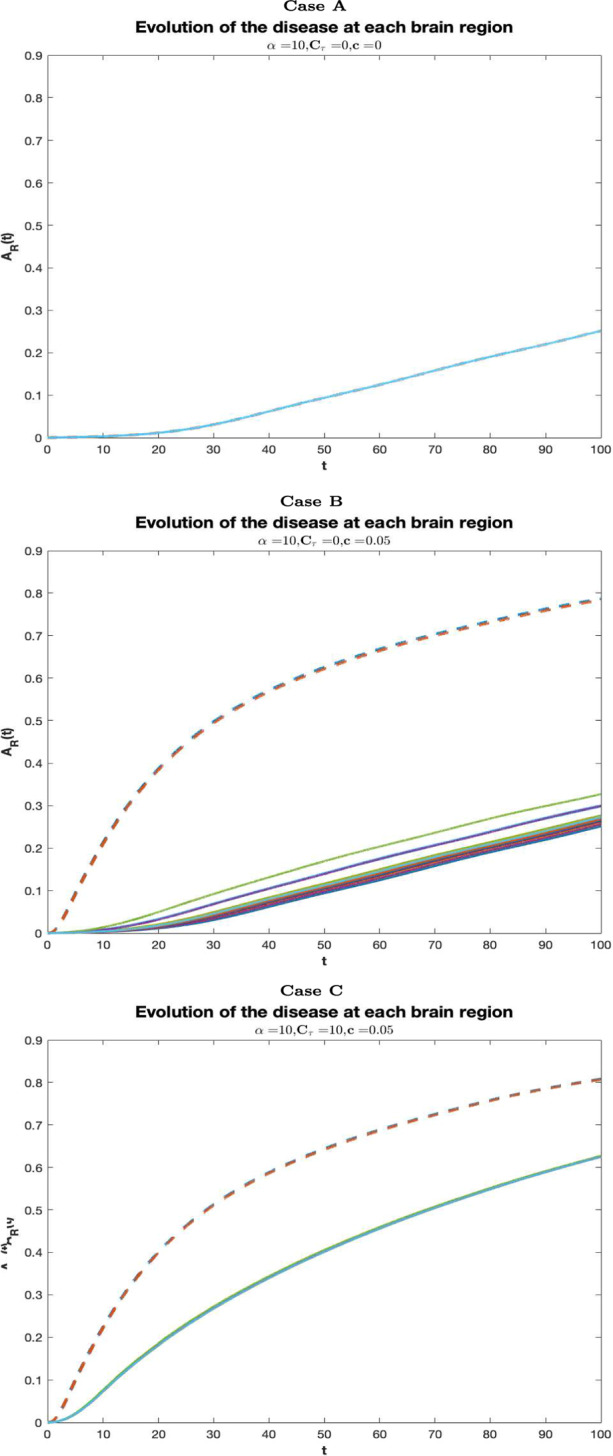
Temporal evolution of the AD at each brain region for $\alpha =10, C_\tau =0, c=0$, (**A**), $\alpha =10, C_\tau =0, c=0.05$, (**B**), $\alpha =10, C_\tau =10, c=0.05$, (**C**). Each curve corresponds to a distinct region. The dashed curves represent the right entorhinal cortex (blue) and the left entorhinal cortex (red) (color figure online)

**Fig. 5:**
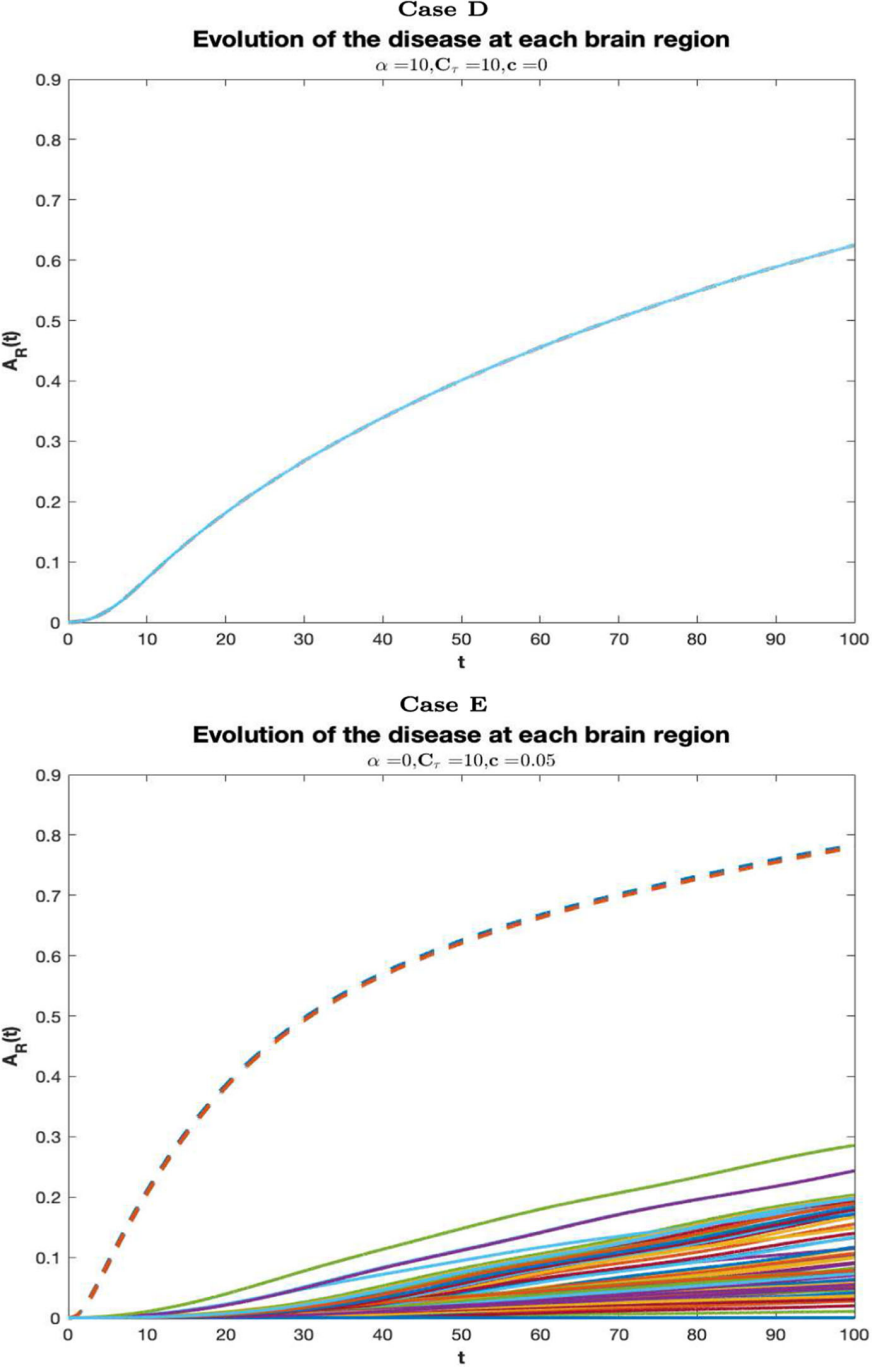
Temporal evolution of the AD at each brain region for $\alpha =10, C_\tau =10, c=0$, (**D**), $\alpha =0, C_\tau =0, c=0.05$, (**E**). Each curve corresponds to a distinct region. The dashed curves represent the right entorhinal cortex (blue) and the left entorhinal cortex (red) (color figure online)

In cases B, C, E the Tau-seeding region (entorhinal cortex) remains the most damaged area, followed by amygdala, hippocampus, temporal pole, isthmuscingulate cortex, insula cortex and parahippocampal cortex although the differences in the evolution of AD between regions are minimal (excluded the seeding zone) in case C and more relevant in cases B and E. In case D, tau seeding at EC is not considered; consistently, the evolution of the disease at EC is not very different from any other cerebral region, suggesting that the effect of this process targets specific brain areas, that are the EC and a few more regions connected with it like amygdala and hippocampus. This fact is more evident in cases B and E, where the tau seeding at EC is the only source for monomeric tau and in addition to EC, the most impaired cerebral zones are those connected to EC, as amygdala and hippocampus, and a few more areas connected with the latter, like, temporal pole, isthmus cingulate cortex, insula cortex and parahippocampal cortex. In Fig. 3 longitudinal graphs of *A*(*t*), describing the evolution of the disease in the whole brain, are shown for each of the cases listed in Table 2. Within the cases considered, the neuronal damage is the most severe in case C (yellow curve), followed with minimal differences by case D (purple curve), indicating, consistently, that the effect of the Gamma-shaped seeding affects certain cerebral regions rather than the whole cerebral network. In cases A (blue curve) and B (red curve), the overall cerebral damage is milder than in cases C and D. This fact is interesting since it expresses the phenomenon that the injury exerted by the A$\beta$ or by both A$\beta$ and Tau when the first does not directly induce the production of the latter, is much less severe than in the case of misfolded tau injection enhanced by A$\beta$. Thus, these observations suggest that A$\beta$ and Tau are more harmful when they act together. The simulation in Fig. 3 may provide support to the fact that the interaction between Tau and A$\beta$ increases the toxicity of both proteins and, combined with the process of Tau seeding, plays a crucial role in brain damaging.

In case E, when monomeric A$\beta$ does not coagulate in longer clusters, the cerebral damage is less serious than in the other cases, confirming once again the central role of A$\beta$ oligomers in neuronal impairment also through their involvement in Tau misfolding. Case E may represent the theoretical action of a drug able to completely inhibit the aggregation of A$\beta$. Although such action seems to be successful in limiting the overall brain’s damage, it is ineffective in controlling the impairment of specific regions (see Fig. 4 case E ) where the disease becomes more and more serious as time goes on.

We observe that in case E summarized in Table 2 we inhibit the agglomeration of A$\beta$ in clusters and consequently, the process of Tau seeding driven by A$\beta$ oligomers is not triggered independently of the value of the constant $C_\tau$.

## Data Availability

Data will be made available on reasonable request.
